# Real-world outcomes of brentuximab vedotin as consolidation therapy after autologous stem cell transplantation in relapsed/refractory Hodgkin lymphoma: A systematic review and meta-analysis

**DOI:** 10.1038/s41409-025-02557-7

**Published:** 2025-04-08

**Authors:** Anna Sureda, Astrid Pavlovsky, Dalah Haidar, Fjoralba Kristo, Vanessa Stache, Athanasios Zomas

**Affiliations:** 1 Institut Català d’Oncologia – Hospital Duran i Reynals, IDIBELL, Universitat de Barcelona, Barcelona, Spain; 2 FUNDALEU Research Center, Buenos Aires, Argentina; 3 Centro de Hematologia Pavlovsky, Buenos Aires, Argentina; 4 Grupo Argentino de Tratamiento de la Leucemia Aguda (GATLA), Buenos Aires, Argentina; 5 Takeda Pharmaceuticals International AG – Singapore Branch, Singapore, Singapore; 6 Takeda Development Center Americas, Inc., Cambridge, MA USA; 7 Takeda Pharmaceuticals International AG, Zürich, Switzerland

**Keywords:** Hodgkin lymphoma, Molecularly targeted therapy

## Abstract

Brentuximab vedotin (BV) as post-autologous stem cell transplantation (ASCT) consolidation was shown to reduce the relapse risk among high-risk patients with relapsed/refractory Hodgkin lymphoma (RRHL) in the clinical trial setting. This systematic review and meta-analysis characterizes real-world evidence (RWE) on the effectiveness and safety of BV as post-ASCT consolidation in 1504 adult and pediatric patients with RRHL from 23 studies across 17 countries. A random-effects model yielded pooled progression-free survival (PFS) and overall survival rates (OS); PFS: 2-year, 74.2%; 5-year, 65.8%; OS: 2-year, 95.8%; 5-year, 91.9%. The most common any-grade adverse events were neuropathy (34.2%) and neutropenia (20.2%). Despite heterogeneity in populations and outcomes, this analysis utilizing real-world data corroborates the efficacy and safety of BV as post-ASCT consolidation in RRHL reported in the experimental arm of the Phase III AETHERA trial. The favorable PFS results in cases exposed to BV prior to ASCT indicate the value of BV in controlling Hodgkin lymphoma (HL) in the salvage setting. Continued research is essential to refine BV treatment strategies amid the evolving treatment landscape.

## Introduction

Hodgkin lymphoma (HL) has a high cure rate, with more than 80% of patients with classic HL achieving long-term remission following first-line therapy [1]. However, 10–30% of patients with advanced-stage disease (IIB–IV) experience relapse after frontline treatment [2–6]. High-dose chemotherapy followed by consolidation with autologous stem cell transplantation (ASCT) is recommended for relapsed or refractory HL (RRHL) [7, 8] and has a cure rate of approximately 50% [9, 10]. Relapse or progression after ASCT typically occurs early, with 71% of patients relapsing within the first year and 91% within the first 2 years [9, 11]. Risk factors for post-ASCT relapse in HL include early relapse ( ≤ 3 months), stage IV disease, poor Eastern Cooperative Oncology Group performance status, bulky disease, extranodal lesions, B symptoms, and nonresponse to salvage chemotherapy (short first complete response [CR] duration or positron emission tomography [PET]-positive residual disease) [9, 12–14].

The Phase III AETHERA trial (2010–2012) established BV as an effective post-ASCT consolidation in HL, significantly improving progression-free survival (PFS) in patients at high risk for post-ASCT relapse or progression [15]. However, this trial excluded patients with prior BV exposure and did not mandate PET evaluations at study initiation. Real-world evidence, derived from real-world data, complements RCTs by providing external validity. Recent real-world studies have described the results of BV as post-ASCT consolidation in pediatric and adult populations with RRHL across multiple regions and countries [16–38]. This systematic literature review and meta-analysis aims to describe and enhance the existing real-world evidence on efficacy and safety outcomes of BV as post-ASCT consolidation or maintenance therapy (as defined within each study) for adult and pediatric patients with RRHL.

## Methods

A systematic review was conducted simultaneously across BIOSIS Previews^®^, Embase^®^, and MEDLINE using ProQuest-Dialog, following a prespecified protocol (PROSPERO, CRD42023471178). In line with best practices, identical searches were conducted on October 10, 2023 and May 02, 2024 using a defined search string (Supplementary Table 1) and covered publications indexed from January 01, 1998 to May 02, 2024. Additional abstracts were retrieved through pragmatic searches of prespecified clinical societies and conference proceedings (2014–2023), selected based on relevance identified during database searches and to capture the latest abstracts not yet indexed. The review adhered to the Preferred Reporting Items for Systematic Reviews and Meta-analysis (PRISMA) guidelines [39].

Real-world observational studies, used here as a general term to describe the included studies, primarily comprised retrospective cohort studies and case series reporting efficacy and safety outcomes in adult and pediatric patients with RRHL treated with BV, either alone or in combination with other therapies, as post-ASCT consolidation or maintenance, were included. Journal articles, congress abstracts, and case series with at least five patients were eligible, with no language restrictions, while clinical trials, systematic reviews, and case reports were excluded. Key outcomes included BV usage patterns, PFS, overall survival (OS), and the most common adverse events (AEs; as reported in the relevant real-world studies). Two independent reviewers screened sources using pre-defined criteria and extracted data from eligible publications in a prespecified extraction table, with conflicts resolved by consensus or a third assessor. Additional information was sought from authors when necessary.

The methodological quality of eligible studies was assessed using the Joanna Briggs Institute critical appraisal tools for cohort studies (11 questions) or case series (10 questions) [40]. Each question was evaluated with one of the following responses: “yes” (criterion met), “no” (criterion unmet), “unclear,” or “not applicable.” To be considered of acceptable methodological quality, the review team prespecified that studies had to meet at least 7 of the criteria for cohort studies (11 questions) or case series (10 questions) [41]. The methodological quality of abstracts could not be ascertained due to insufficient information.

### Meta-analysis

Combined analyses of data from journal articles and conference abstracts were performed for all outcomes. In some studies, outcomes of interest were not reported, requiring data assumptions and/or calculations as part of the data analysis (Supplemental Methods). Study and patient characteristics were documented for each study included. In studies where only a subset of patients met the inclusion criteria, patient characteristics were documented solely for that subset, where available. Continuous variables were reported as medians and ranges, whereas categorical variables were reported as frequencies and percentages.

The DerSimonian and Laird random-effects method was used to pool estimates, regardless of the degree of heterogeneity between the study results. In the analyses, studies were weighted by the standard error of the outcome metric.

For binary outcomes (PFS and OS rates), the proportions of patients meeting the outcomes were pooled. Before pooling, Freeman–Tukey double arcsine transformation was performed to stabilize the variances when the proportions are close to zero and one, and a normal approximation to the binomial distribution does not hold.

The frequency of AEs was presumed to follow a Poisson distribution. The mean number of occurrences per patient was computed, with each study weighted by the standard error of the mean value.

Heterogeneity between studies was evaluated by considering both the significance of between-study heterogeneity and the magnitude of the *I²* value. Substantial heterogeneity was inferred if the *I²* value exceeded 50%. Heterogeneity was not analyzed for outcomes from two or fewer studies. All statistical analyses were performed using Stata version 15.1.

Forest plots presented outcomes for individual studies alongside the pooled results. Weights for individual studies were assigned based on their contribution to the pooled estimates, calculated as the inverse of the variance of the treatment effect.

## Results

**Fig. 1:**
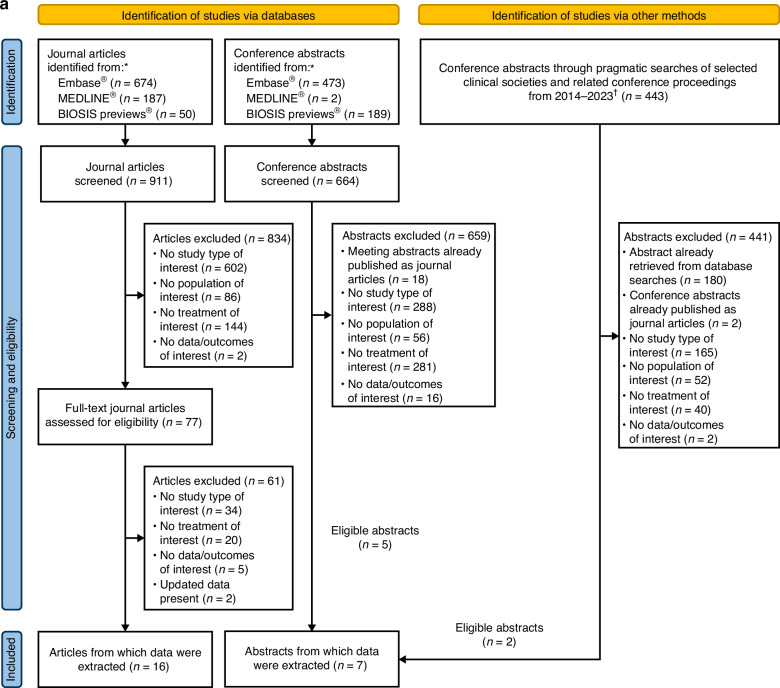

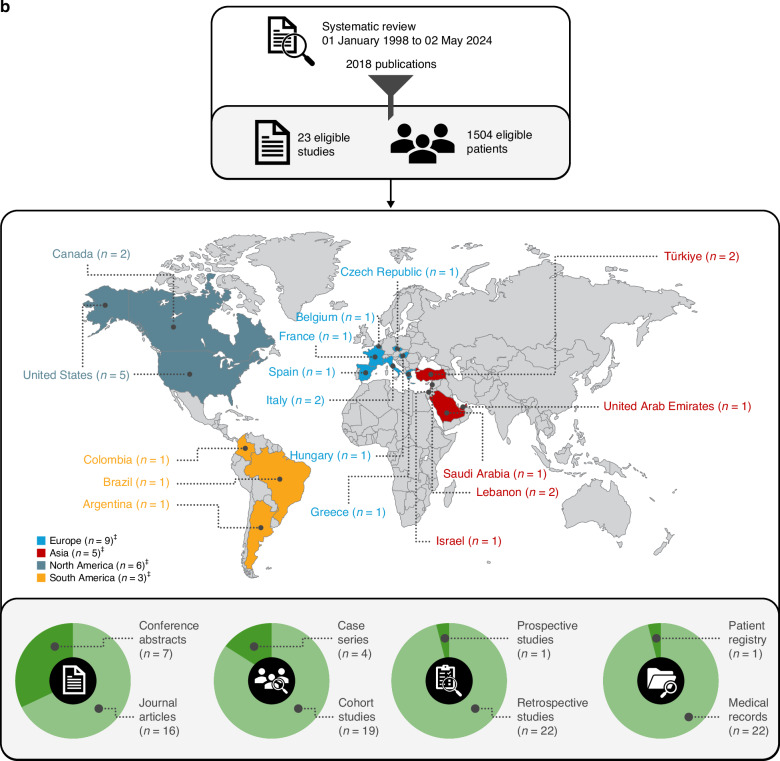
Systematic literature review process. **a** PRISMA flowchart of search results; **b** Study characteristics. *Duplicates removed. ^†^Selected congresses: The American Society of Hematology (ASH) Annual Meeting and Exposition, European Hematology Association (EHA) Annual Congress, American Society of Clinical Oncology (ASCO) Annual Meeting, European Society for Medical Oncology (ESMO) Congress, International Symposium on Hodgkin Lymphoma (ISHL), International Conference on Malignant Lymphoma (ICML), British Society of Haematology (BSH) Annual Scientific Meeting, Society of Hematologic Oncology (SOHO) Annual Meeting, International Society for Pharmacoeconomics and Outcomes Research (ISPOR) European Congress. ^‡^Some studies had patient data from more than one country. PRISMA: Preferred Reporting Items for Systematic Reviews and Meta-analysis.

Of 911 journal articles and 664 conference abstracts from electronic database searches and 443 abstracts from relevant conference proceedings, 16 journal articles [16–31] and 7 conference abstracts [32–38] were considered eligible for data extraction. A PRISMA flowchart outlines the reasons for study exclusion (Fig. 1a).

Study characteristics are presented in Table 1. Data were extracted from 1504 eligible patients with HL in 23 studies from 17 countries (Fig. 1b). Of these studies, 22 were retrospective, and 1 was prospective. Medical records were the data source in 22 studies, while one utilized a patient registry. Most studies (*n* = 15) scheduled the administration of 16 BV cycles as post-ASCT consolidation, per the approved indication.

**Table 1:** Study characteristics.

Study	Study region	Study design	Study period	Patients, n^a^	Data source	Post-ASCT BV consolidation/maintenance cycles planned, *n*	Follow-up period, months, median (range)	Response assessment method	Effectiveness and safety parameters
**Articles**									
Flerlage [16]	US	Retrospective single center study	NA	5	Medical records	16	NA (29–76)	NA	CR, OS, AE
Sakellari [17]	Greece	Retrospective single center study	NA	10	Medical records	16^b^	34.3 (1.5–202.2)	PET and CT scans according to the 2007 IWG revised response criteria for malignant lymphoma	ORR, AE
Taçyıldız [18]	Türkiye	Retrospective single center study	July 2012–August 2017	8	Medical records	12	34 (12–42)	PET scans	AE
Kort [19]	Lebanon	Retrospective, single center study	2014–2018	20	Medical records	4	26.5 (5–50)	PET-CT scans	OS, PFS, AE
Akay [20]	Türkiye	Retrospective multicenter study	January 2016–July 2019	75	Medical records	16	26 (6–55)	PET-CT or CT scans according to the 2007 IWG revised response criteria for malignant lymphoma	CR, OS, PFS, AE
Fernandez [21]	US	Retrospective single center study	January 2015–December 2017	6	Medical records	16	23 (13–30)	FDG PET and CT scans	PFS, AE
Kedmi [22]	Israel	Retrospective single center study	2015–2020	14	EMRs	16	19.1 (NA)	PET-CT scans according to the 2014 Lugano response criteria	CR, PR, AE
Marouf [23]	France	Retrospective multicenter study	2012–2017	115	Medical records	16	35 (NA)	FDG PET scan – as reported by 2 independent physicians	OS, PFS, AE
Massano [24]	Italy	Retrospective multicenter study	January 2020–December 2020	27	Medical records	NA	33.6 (7.2–106.8)	PET or CT scans according to the 2014 Lugano response criteria	PD
Massaro [25]	Belgium	Retrospective single center study	May 2019–October 2021	6	Medical records	16	24.75 (17.4–29.9)	PET-CT scan according to the 2014 Lugano response criteria and 2016 Refinement of the Lugano Classification lymphoma response criteria	CR, PR, OS, AE
Massaro [26]	Italy	Retrospective multicenter study	April 2011–August 2020	105	Medical records	16	20 (2–108)	PET-CT or CT scans according to the 2014 Lugano response criteria – as reported by treating physician	CR, PR, OS, PFS, AE
Forlenza [27]	US, Canada	Retrospective multicenter study	January 2014–January 2021	67	Medical records	16	37 (3–75)	FDG PET scans	OS, PFS, AE
Husi [28]	Hungary	Retrospective multicenter study	1 January 2016–31 December 2020	108^c^	Medical records	NA	39 (1–76)	PET-CT scans according to the 2016 Refinement of the Lugano Classification lymphoma response criteria	OS, PFS
Martinez [29]	Spain	Retrospective multicenter study	May 2015–March 2021	62	Medical records	16^b^	56.4 (50.4–63.6)	PET-CT scans according to the 2014 Lugano response criteria	PFS, AE
Wagner [30]	US	Retrospective multicenter study	1 July 2015–30 June 2019	118	Medical records	16	35.5 (NA)	FDG PET-CT or CT scans according to the 2014 Lugano response criteria	PFS, AE
Damlaj [31]	Saudi Arabia, UAE, Lebanon	Retrospective multicenter study	2010–2020	35	Medical records	16	19.3 (1–89.2)	PET-CT scans	OS, PFS, AE
**Abstracts**									
Aragão [32]	Brazil	Retrospective single center study	2011–2016	8	Medical records	16	14.3 (NA)	NA	CR, PR, OS, AE
Patiño [33]	Colombia	Retrospective multicenter study	NA	53	Medical records	NA	23.5 (4.2–133.2)	PET-CT or CT scans according to the Lugano response criteria; uCR according to the 2007 IWG revised response criteria for malignant lymphoma	OS, PFS, AE
Chung [34]	Canada	Retrospective study	July 2011–June 2020	49	Medical records	NA	62.2 (NA)	NA	AE
Michalka [35]	Czech Republic	Retrospective multicenter study	January 2015–December 2021	39	Medical records	16	28 (NA)	NA	CR, OS, PFS, AE
Munoz [36]	Europe	Retrospective study	May 2016–January 2021	309	Registry	16^b^	20 (NA)	NA	OS, PFS
Falade [37]	US	Retrospective multicenter study	2010–2022	224	Medical records	NA	58.8 (NA)	PET scans	OS, PFS
Fiad [38]	Argentina	Prospective multicenter study	May 2021–July 2023	41	Prospective data	NA	13.1 (NR)	FDG PET scans	AE

^a^1504 of 2568 patients met the inclusion criteria.

^b^As previously reported in AETHERA study.

^c^Exact number of eligible patients not reported. 18 of 126 patients did not receive post-ASCT BV treatment.

*AE* adverse event, *ASCT* autologous stem cell transplantation, *BV* brentuximab vedotin, *CR* complete response, *CT* computed tomography, *EMR* electronic medical record, *FDG* fluorodeoxyglucose, *IWG* International Working Group, *NA* not available, *ORR* overall response rate, *OS* overall survival, *PET* positron emission tomography, *PFS* progression-free survival, *PR* partial response, *uCR* unconfirmed complete response, *UAE* United Arab EMirates, *US* United States.

Patient characteristics are summarized in Table 2 and Supplementary Table 2. Of the 23 publications, 10 included pediatric patients. Administration of pre-ASCT BV, either alone or in combination with other salvage agents as a bridge to transplant, was reported in 50.5% of all eligible patients from 11 studies. Three studies reported administering a median number of 4 or 5 BV cycles pre-ASCT.

**Table 2:** Patient characteristics for included studies.

Study	Age, years, median (range)	Males, %	Prior therapies, median (range)	BV cycles prior to ASCT, median (range)	BV cycles post ASCT, median (range)	Stage III–IV disease/ Advanced stage, %	Pre-ASCT positive PET status, %
**Articles**							
Flerlage [16]	17 (16–22)^a^	20	NA	NA	16 (4–16)	NA	NA
Sakellari [17]	NA^b^	NA^b^	NA	NA	NA	NA	NA
Taçyıldız [18]	14 (6–18)	88	NA	4 (4–8)	8 (4–8)	100	NA
Kort [19]	26 (18–61)^c^	40	3 (2–5)	NA	4 (3–4)	60	6
Akay [20]	31 (18–65)^c^	56	NA	NA	NA	NA	57
Fernandez [21]	15 (12–18)^d^	17	NA	NA	16 (12–16)	83.3	NA
Kedmi [22]	32.5 (21–68)^e^	36	NA	NA	12 (2–20)	78.6	NA
Marouf [23]	34 (16–68)	54	NA	NA	11 (3–18)	58	NA
Massano [24]	NA^b^	NA^b^	NA	NA	NA	NA	NA
Massaro [25]	NA^b^	NA^b^	3 (2–3)	5 (2–7)	7 (1–14)	NA	NA
Massaro [26]	33 (18–68)	56	2	4 (2–11)	10 (2–16)	44	NA
Forlenza [27]	17 (8–21)^f^	49	1 (1–3)	NA	NA	49	31
Husi [28]	NA^b^	NA^b^	NA	NA	NA	NA	NA
Martinez [29]	35 (16–70)^c^	52	2 (1–6)	NA	14 (2–16)	51.6	24
Wagner [30]	36 (27–42)	53	NA	NA	12 (2–25)	70	NA
Damlaj [31]	NA^b^	NA^b^	NA^b^	NA	16 (3–16)	NA^b^	NA^b^
**Abstracts**							
Aragão [32]	26	NA	NA	NA	12 (4–16)	NA	NA
Patiño [33]	29 (17–66)^c^	51	3 (2–7)	NA	11 (1–16)	NA	NA
Chung [34]	NA^b^	NA^b^	NA	NA	10.5 (1–16)	NA	NA
Michalka [35]	37 (19–65)	NA	NA	NA	8 (1–16)	~80	NA
Munoz [36]	31 (18–70)	52	2 (1–6)	NA	NA	NA	NA
Falade [37]	NA^b^	NA	NA	NA	10 (1–18)	NA	NA
Fiad [38]	NA^b^	NA^b^	NA	NA	NA	NA^b^	NA

^a^Age at consolidation initiation.

^b^Data not reported separately for patients receiving BV as post-ASCT consolidation (study inclusion criteria).

^c^Age at transplant.

^d^Age at diagnosis.

^e^Age at BV treatment initiation.

^f^Age at relapse.

*ASCT* autologous stem cell transplantation, *BV* brentuximab vedotin, *NA* not available, *PET* positron emission tomography.

Pre-ASCT CR rates, evaluated using PET-CT or CT, were reported in 12 studies and ranged from 28.3% to 100% in all eligible patients. Treatment response before ASCT was evaluated using PET-CT or CT in 18 studies, with assessments based on the 2016 Lugano Classification (*n* = 8) or 2007 revised response criteria for malignant lymphoma from the International Working Group (*n* = 3). Five studies did not report the response assessment method.

All studies, with the exception of one [24], utilized BV as a single-agent. Eighteen studies defined the administration of BV as post-ASCT consolidation [16–22, 24–27, 29, 32–36, 38], while five studies defined administration as post-ASCT maintenance [23, 28, 30, 31, 37]. The dosing regimen was 1.8 mg/kg every 3 weeks in 12 studies. Sixteen studies reported administering a median number of BV cycles between 4 and 16 following ASCT.

### Survival outcomes

**Fig. 2:**
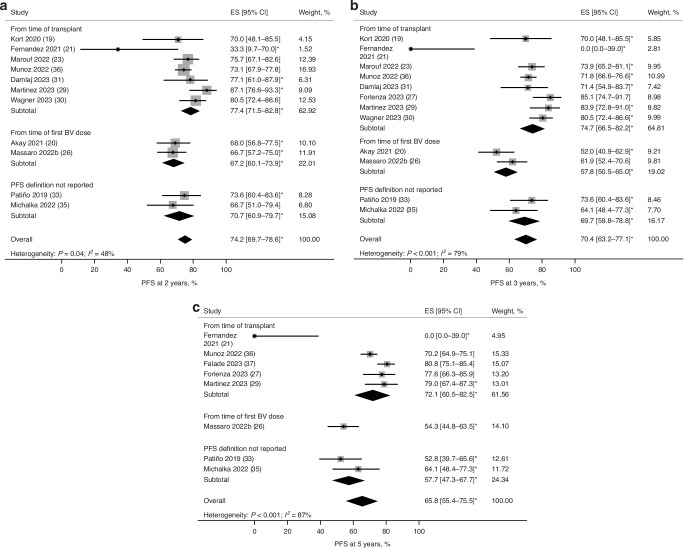
Pooled estimates of PFS rates at 2, 3, and 5 years of follow-up in all patients based on PFS definitions. **a** PFS rates at 2 years of follow-up. **b** PFS rates at 3 years of follow-up. **c** PFS rates at 5 years of follow-up. ^*^PFS rates were estimated using Kaplan–Meier curves. ^†^Heterogeneity was not computed for outcomes with data provided by only 1 or 2 studies. BV brentuximab vedotin, CI confidence interval, ES effect size, NC not calculated, PFS progression-free survival.

PFS estimates were analyzed based on definitions that varied across studies (Fig. 2; Supplementary Fig. 1). In studies reporting PFS, ten calculated it post-ASCT, two post-BV consolidation, and two did not provide a definition. Pooled 2- and 5-year PFS were 74.2% (95% CI: 69.7–78.6; Fig. 2a) and 65.8% (95% CI: 55.4–75.5; Fig. 2c), respectively, with considerable heterogeneity between studies. Husi et al. reported 5-year PFS of 69% with BV as post-ASCT consolidation and 70% for patients receiving BV as both salvage therapy and post-ASCT consolidation. However, this study was excluded from the 5-year PFS rate estimation due to unclear patient numbers for BV as post-ASCT consolidation [28].

**Fig. 3:**
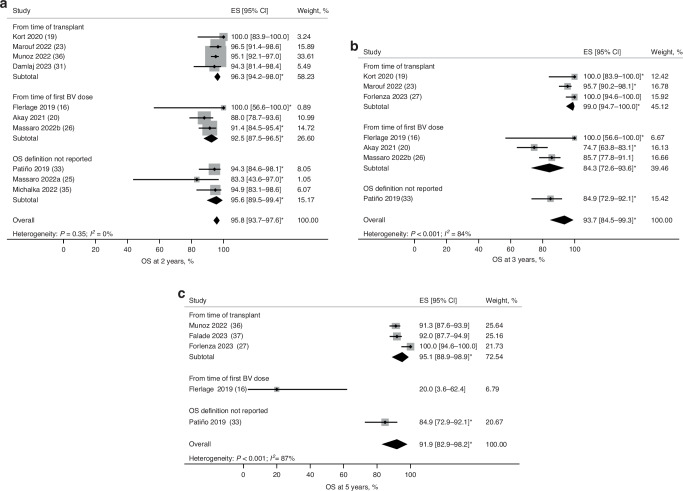
Pooled estimates of OS rates at 2, 3, and 5 years of follow-up in all patients based on OS definition. **a** OS rates at 2 years of follow-up. **b** OS rates at 3 years of follow-up. **c** OS rates at 5 years of follow-up. *OS rates were estimated using Kaplan–Meier curves. BV brentuximab vedotin, CI confidence interval, ES effect size; NC not calculated, OS overall survival.

OS estimates were analyzed based on definitions that varied across studies (Fig. 3; Supplementary Fig. 2). In studies reporting OS, seven calculated it post-ASCT, three post-BV consolidation, and four studies did not provide a definition. Pooled 2- and 5-year OS were 95.8% (95% CI: 93.7–97.6; Fig. 3a) and 91.9% (95% CI: 82.9–98.2; Fig. 3c), respectively. There was minimal heterogeneity between studies reporting 1- and 2-year OS rates. Husi et al. reported 5-year OS of 89% with BV as post-ASCT consolidation and 93% for patients receiving BV as both salvage therapy and post-ASCT consolidation. However, this study was excluded from the 5-year OS rate estimation due to unclear patient numbers for BV as post-ASCT consolidation [28].

**Fig. 4:**
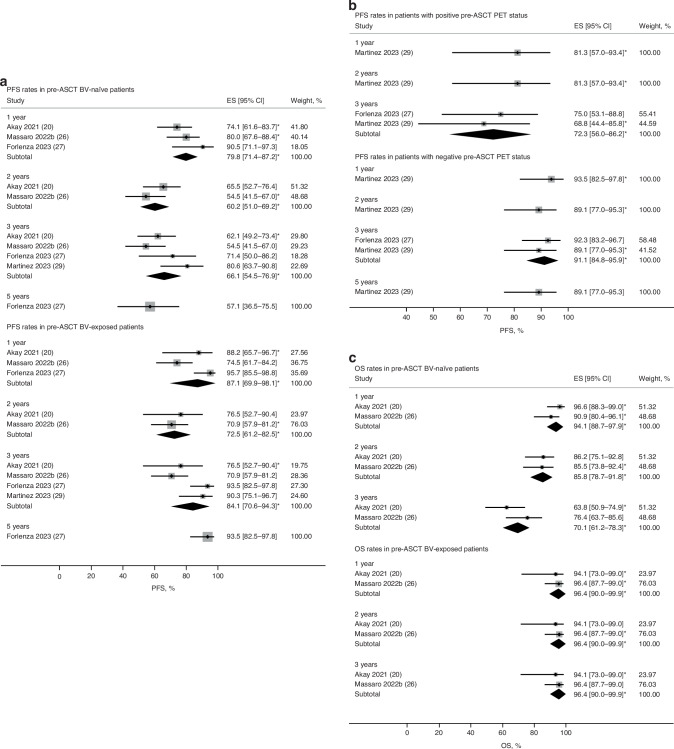
Pooled estimates of survival rates in patient subgroups at different time points during follow-up. **a** PFS rates in BV-naïve and BV-exposed patients; **b** PFS rates in patients with positive and negative PET status; **c** OS rates in BV-naïve and BV-exposed patients. ASCT autologous stem cell transplantation, BV brentuximab vedotin, CI confidence interval, ES effect size, OS overall survival, PET positron emission tomography, PFS progression-free survival.

Pooled estimates of PFS and OS rates at various follow-up time points were obtained for patient subgroups where reported. Patients who received BV as salvage therapy pre-ASCT (BV-exposed patients) had improved 2- and 5-year PFS compared with BV-naïve patients: 72.5% vs. 60.2% and 93.5% vs. 57.1%, respectively (Fig. 4a; Supplementary Table 3). Patients with negative pre-ASCT PET status appeared to have a higher 2- and 3-year PFS (89.1% and 91.1%, respectively) than in those with positive PET status (81.3% and 72.3%, respectively) (Fig. 4b; Supplementary Table 3). Improved 3-year OS was observed in BV-exposed patients (96.4%) compared with BV-naïve patients (70.1%; Fig. 4c; Supplementary Table 3).

Relapse during or after post-ASCT BV consolidation or maintenance was reported in 7 studies and ranged from 0% to 29% of all eligible patients [18, 19, 23, 26, 29, 33, 35]. Disease progression was reported in 11 studies and ranged from 6.5% to 33.3% of all eligible patients [19, 20, 22, 25, 26, 29, 30, 32, 34, 35, 38].

### Safety outcomes

**Fig. 5:**
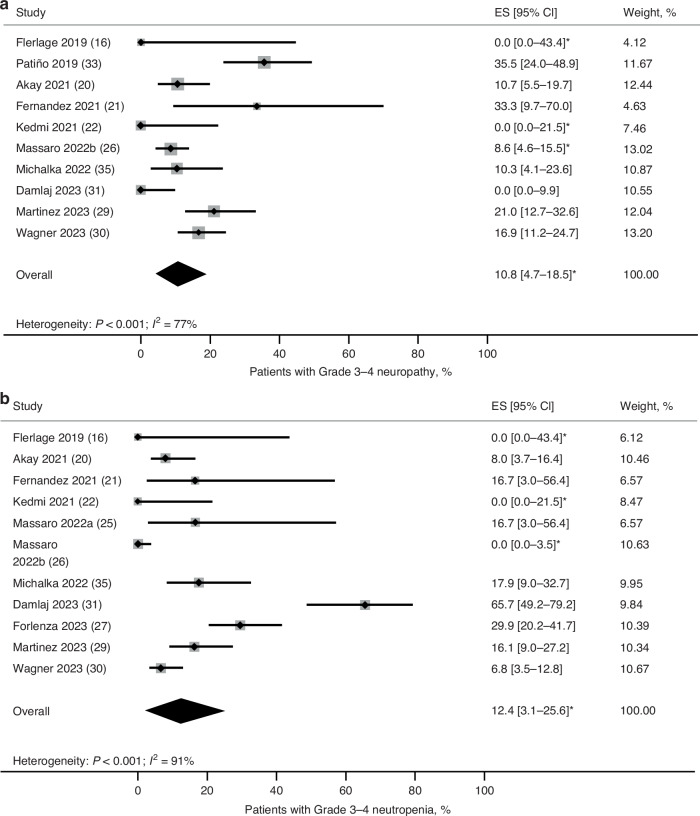
Pooled estimates of AEs in patients with RRHL treatment with BV as post-ASCT consolidation. **a** Incidence of Grade 3–4 neuropathy. **b** Incidence of Grade 3–4 neutropenia. *Estimated. AE adverse event, ASCT autologous stem cell transplantation, BV brentuximab vedotin, CI confidence interval, ES effect size, NC not calculated, RRHL relapsed/refractory Hodgkin lymphoma.

Nineteen studies reported safety data with BV as post-consolidation in patients with RRHL. Of these, 5 studies used the National Cancer Institute Common Terminology Criteria for Adverse Events, while 14 did not specify a grading scale. The pooled mean number of any grade AEs per patient was 0.42 and the pooled mean number of Grade 3–4 AEs per patient was 0.08 (Supplementary Fig. 3a, b). The proportion of patients with any grade AEs was 47% and with Grade 3–4 AEs was 6.2% (Supplementary Fig. 3c, d). The most common any grade AEs reported were neuropathy (34.2% [95% CI: 21.8–47.6]) and neutropenia (20.2% [95% CI: 7.6–36.2]; Supplementary Table 4; Supplementary Fig. 3e, f). Grade 3–4 neuropathy and neutropenia occurred in 10.8% (95% CI: 4.7–18.5; Fig. 5a) and 12.4% (95% CI: 3.1–25.6; Fig. 5b) of patients, respectively.

## Discussion

This systematic review and meta-analysis examined 23 real-world observational studies that reported the effectiveness and/or safety of BV as post-ASCT consolidation in patients with RRHL. Over half of these studies followed BV dosing regimens similar to those used in the Phase III AETHERA trial [15, 42] and the BV prescribing information [43]. In line with AETHERA [15, 42], some studies included patients with at least one high-risk feature of HL, such as primary refractory HL, initial remission duration of <12 months, partial response or stable disease to most recent salvage therapy, extranodal disease at relapse, B symptoms at relapse, or ≥2 prior salvage therapies. However, AETHERA excluded patients with prior BV exposure.

In this systematic review and meta-analysis, estimated PFS at 2- and 5-years were 74% and 66%, respectively. These results align with the findings from AETHERA of improved PFS (2- and 5-year PFS of 63% and 59%, respectively), when compared with placebo [15, 42]. Nevertheless, a direct comparison is challenging due to varying patient characteristics in the real-world clinical setting. In the included studies, where available, the proportion of patients with primary refractory HL ranged from 7% to 79%, while in AETHERA, 60% of evaluated patients were deemed primary refractory [15]. Our analysis estimated high OS rates, with trends persisting for up to 11 years. Patients who achieve a response to salvage treatment prior to ASCT, particularly a CR, are likely to have better survival rates compared with those who did not achieve a response. In 12 studies, 28.3–100% of patients achieved CR prior to ASCT, which to a certain extent may have contributed to improved survival rates.

Our analysis reported improved PFS and OS rates in patients with pre-ASCT BV exposure compared with those who were BV-naïve. This is in keeping with the conclusions from the European Society for Blood and Marrow Transplantation retrospective analysis of post-ASCT BV use, presented at the 2024 American Society of Hematology Annual Meeting [44]. While a selection bias of patients receiving pre-ASCT BV therapy cannot be excluded, this observation suggests a potential therapeutic advantage of prior BV therapy. BV exposure may enhance the elimination of the malignant clone or modify the tumor microenvironment, promoting immune-mediated clearance of tumor cells and thereby enhancing the effectiveness of subsequent treatments such as immunotherapy or salvage chemotherapy and effective bridging to ASCT [45, 46]. Additionally, BV has lower reported systemic toxicity compared with traditional non-targeted chemotherapy, thus improving overall treatment tolerability and potentially enabling more aggressive interventions [47, 48].

The impact of achieving a PET negative CR could not be assessed in the AETHERA trial as PET-CT was not mandatory. Our analysis reported higher PFS rates in patients with negative pre-ASCT PET-CT status compared with those with positive pre-ASCT PET-CT status, highlighting the prognostic value of routine pre-ASCT PET-CT assessment. A Phase II trial involving 105 patients with HL reported improved event-free survival in those with negative versus positive pre-ASCT PET-CT status, highlighting imaging as a key tool for assessing response to salvage therapy and guiding subsequent treatment decisions [49]. Since then, additional studies have demonstrated the benefit of achieving negative PET-CT status before ASCT [50, 51].

In AETHERA, patients received a median of 15 BV cycles (range: 1–16) once every 3 weeks [15]. Our analysis of real-world studies reported a range of 1 to 16 post-ASCT BV cycles, with one study utilizing 4 cycles. However, it was not feasible to calculate the median number of BV cycles due to the lack of individual patient data. The lower number of BV cycles utilized in some real-world studies may have been influenced by poor treatment tolerability in patients, potentially resulting in a lower reporting of AEs. Neuropathy and neutropenia were the most frequently reported AEs in both this analysis and AETHERA [15]; however, the incidence was substantially lower in this analysis (neuropathy, 34.2% vs. 56%; neutropenia, 20.6% vs. 35%). This difference should be interpreted with caution due to the reliance of real-world studies on data from routine clinical practice, where AE reporting may be less rigorous compared with controlled trial settings dedicated to safety monitoring. Additionally, inconsistencies are common in AE documentation within electronic health records and claims databases used for real-world studies, contributing to the underreporting of AEs.

This analysis has inherent limitations common to meta-analyses, emphasizing caution in data interpretation. Literature research was limited to specific databases and nine pre-determined conferences, and the retrospective nature of most studies limits causal conclusions and control of confounders. The methodological quality of abstracts could not be determined due to insufficient information. In studies where only a subset of patients met the inclusion criteria, patient demographics and disease characteristics were often inadequately reported for the patient group of interest. Adult and pediatric patients could not be analyzed separately due to a lack of distinct data, potentially masking efficacy and toxicity differences. Variations in outcome definitions across studies, and incomplete reporting of numerical data for specific outcomes, necessitated assumptions and/or calculations during data analysis, potentially introducing sources of bias and uncertainty. A key challenge in this analysis is the limited availability of detailed and clear patient data, restricting the ability to conduct subgroup analyses despite the large cohort size. This limitation is particularly significant given the heterogeneous nature of the patient population, hindering analysis of important variables, such as the role of BV consolidation in patients with negative pre-ASCT PET-CT status, the potential benefit of post-ASCT BV consolidation in patients previously exposed to BV, and the efficacy of a shortened consolidation regimen. To address these challenges, future studies should prioritize the collection of comprehensive and high-quality patient data, enabling more granular analysis of treatment effects. Although BV consolidation therapy holds promise, ongoing vigilance and adaptation to the evolving treatment landscape is crucial in optimizing patient care and improving clinical outcomes. Further research is warranted to address these limitations and provide more robust evidence for clinical decision-making.

Despite heterogeneity in study populations and outcomes, the present analysis reaffirms the effectiveness and safety of BV as post-ASCT consolidation in patients with RRHL in real-world clinical practice, with comparable results to the experimental arm of the AETHERA trial. Based on real world data spanning a decade across multiple countries, our findings highlight the importance of BV consolidation in optimizing treatment outcomes and its robustness across diverse patient populations.

## Supplementary information

Supplemental material

## Data Availability

The datasets, including the redacted study protocol, redacted statistical analysis plan, and study report supporting the results reported in this article, will be made available within 3 months from initial request to researchers who provide a methodologically sound proposal. The data will be provided after their de-identification, in compliance with applicable privacy laws, data protection, and requirements for consent and anonymization.
