# Supplementary material for: Real-world outcomes of brentuximab vedotin as consolidation therapy after autologous stem cell transplantation in relapsed/refractory Hodgkin lymphoma: A systematic review and meta-analysis
Source: Bone Marrow Transplant. 2025 Apr 8;60(6):820–31. doi: 10.1038/s41409-025-02557-7 (PMC12151861; doi:10.1038/s41409-025-02557-7)
Supplement: Supplementary file 1 — Supplemental material [file 41409_2025_2557_MOESM1_ESM.docx]

**Real-world outcomes of brentuximab vedotin as consolidation therapy after autologous stem cell transplantation in relapsed/refractory Hodgkin lymphoma: A systematic review and meta-analysis**

**Supplementary online material**

**Supplementary methods**

**Pragmatic searches of conference proceedings**

To identify any recent studies for which there are currently no full publications, we searched proceedings from the following conferences, that took place between 2014 and 2023 (till 01 December 2023):

- The American Society of Hematology (ASH) Annual Meeting and Exposition
- European Hematology Association (EHA) Annual Congress
- American Society of Clinical Oncology (ASCO) Annual Meeting
- European Society for Medical Oncology (ESMO) Congress
- International Symposium on Hodgkin Lymphoma (ISHL)
- International Conference on Malignant Lymphoma (ICML)
- British Society of Haematology (BSH) Annual Scientific Meeting
- Society of Hematologic Oncology (SOHO) Annual Meeting
- International Society for Pharmacoeconomics and Outcomes Research (ISPOR) European Congress

**Search terms and search strings**

The search terms and search strings were developed based on the population, intervention, comparator, and outcome (PICO) framework and the research question. Two search strings were developed for the systematic searches – the first for obtaining relevant journal articles and the second for obtaining relevant conference abstracts. Search terms to exclude case reports, errata, and retractions were incorporated into the search strings (**Supplementary Table 1**).

**Supplementary Table 1** Search terms and search strings

|  | **PICO** | **Set#** | **Search terms** |
| --- | --- | --- | --- |
| **P** | Adult and pediatric patients with Hodgkin lymphoma | S1 | MESH.EXACT("Hodgkin Disease") |
|  |  | S2 | EMB.EXACT.EXPLODE("Hodgkin disease") |
|  |  | S3 | ("cHL") |
|  |  | S4 | (EMB.EXACT("refractory disease") OR EMB.EXACT("refractory cancer") OR EMB.EXACT("refractory tumor")) AND EMB.EXACT.EXPLODE("Hodgkin disease") |
|  |  | S5 | (relapsed OR refractory) AND (Hodgkin OR Hodgkins OR "Hodgkin's") AND (disease OR lymphoma) |
|  |  | S6 | (EMB.EXACT("refractory disease") OR EMB.EXACT("refractory cancer") OR EMB.EXACT("refractory tumor")) AND EMB.EXACT.EXPLODE("classical Hodgkin lymphoma") |
|  |  | S7 | (relapsed OR refractory) AND (classical) AND (Hodgkin OR Hodgkins OR "Hodgkin's") AND (disease OR lymphoma) |
|  |  | S8 | ("RRHL") |
|  |  | S9 | ("R/R HL") |
|  |  | S10 | ("relapsed/refractory cHL") |
|  |  | S11 | ("R/R cHL") |
|  |  | **S12** | **S11 OR S10 OR S9 OR S8 OR S7 OR S6 OR S5 OR S4 OR S3 OR S2 OR S1** |
| **AND** | | | |
| **I** | Brentuximab vedotin consolidation | S13 | ("brentuximab vedotin") |
|  |  | S14 | MESH.EXACT("brentuximab vedotin") |
|  |  | S15 | EMB.EXACT("brentuximab vedotin") |
|  |  | S16 | ("BV") |
|  |  | S17 | ("Bv") |
|  |  | S18 | (EMB.EXACT("consolidation therapy") OR EMB.EXACT("consolidation chemotherapy")) AND EMB.EXACT("brentuximab vedotin") |
|  |  | S19 | MESH.EXACT("Consolidation Chemotherapy") AND MESH.EXACT("brentuximab vedotin") |
|  |  | S20 | (consolidat*) AND ("brentuximab vedotin") |
|  |  | S21 | (consolidative) AND ("brentuximab vedotin") |
|  |  | S22 | EMB.EXACT("monotherapy") AND EMB.EXACT("brentuximab vedotin") |
|  |  | S23 | (monotherapy) AND ("brentuximab vedotin") |
|  |  | S24 | (EMB.EXACT("maintenance therapy") OR EMB.EXACT("maintenance drug dose") OR EMB.EXACT("maintenance chemotherapy")) AND EMB.EXACT("brentuximab vedotin") |
|  |  | S25 | MESH.EXACT("Maintenance Chemotherapy") AND MESH.EXACT("brentuximab vedotin") |
|  |  | S26 | (maintenance) AND ("brentuximab vedotin") |
|  |  | S27 | (Adcetris) |
|  |  | **S28** | **S27 OR S26 OR S25 OR S24 OR S23 OR S22 OR S21 OR S20 OR S19 OR S18 OR S17 OR S16 OR S15 OR S14 OR S13** |
| **AND** | | | |
|  | Autologous stem cell transplant | S29 | MESH.EXACT("Stem Cell Transplantation") |
|  |  | S30 | MESH.EXACT("Hematopoietic Stem Cell Transplantation") |
|  |  | S31 | EMB.EXACT("stem cell transplantation") |
|  |  | S32 | EMB.EXACT("hematopoietic stem cell transplantation") |
|  |  | S33 | EMB.EXACT("autologous hematopoietic stem cell transplantation") |
|  |  | S34 | EMB.EXACT("patient history of stem cell transplantation") |
|  |  | S35 | ("stem cell transplant*" AND (autologous OR haematopoietic OR hematopoietic)) |
|  |  | S36 | ("SCT" AND (auto" OR autologous OR haematopoietic OR hematopoietic)) |
|  |  | S37 | (("HCT" OR "HSCT" OR "SCT") AND ("auto" OR autologous OR post)) |
|  |  | S38 | ("AHCT" OR "ASCT" OR "HCT" OR "HSCT" OR "SCT") |
|  |  | S39 | ("autoHCT" OR "autoHSCT" OR "autoSCT") |
|  |  | S40 | ("postASCT" OR "postHCT" OR "postHSCT") |
|  |  | **S41** | **S40 OR S39 OR S38 OR S37 OR S36 OR S35 OR S34 OR S33 OR S32 OR S31 OR S30 OR S29** |
|  | | **S42** | **S41 AND S28 AND S12** |
| **Search terms to exclude case reports, errata, and retractions** | | S43 | RTYPE("Case Reports") |
|  |  | S44 | EMB.EXACT("case report") |
|  |  | S45 | EMB.EXACT("single-case study") |
|  |  | S46 | MESH.EXACT("Case Reports as Topic") |
|  |  | S47 | MESH.EXACT("Single-Case Studies as Topic") |
|  |  | S48 | RTYPE("Erratum") |
|  |  | S49 | RTYPE("Published Erratum") |
|  |  | S50 | RTYPE("Retraction of Publication") |
|  |  | S51 | RTYPE("Retracted Publication") |
|  |  | S52 | EMB.EXACT("retraction notice") |
|  |  | **S53** | **S52 OR S51 OR S50 OR S49 OR S48 OR S47 OR S46 OR S45 OR S44 OR S43** |
|  |  | **S54** | **S42 NOT S53** |
| **Search terms for conference abstracts** | | S55 | RTYPE("Conference Abstract") |
|  |  | S56 | RTYPE("Meeting") |
|  |  | S57 | PSTYPE("Meeting") |
|  |  | S58 | DTYPE("Conference Abstract") |
|  |  | S59 | DTYPE("Conference") |
|  |  | **S60** | **S59 OR S58 OR S57 OR S56 OR S55** |
| **Search string for conference abstracts** | | **S61** | **S54 AND S60 [meeting abstracts]** |
| **Search string for journal articles** | | **S62** | **S54 NOT S60 [journal articles]** |

**Key:**

Wildcard/truncation: asterisk, question mark, dollar sign

"": double quotation marks, exact phrase

**Data assumptions/calculations**

Some studies did not specifically report data for some outcomes of interest. In these cases, data assumptions and/or calculations were made to assist in the data analysis:

- In cases where overall survival (OS) and progression-free survival (PFS) rates were not reported at specific follow-up time points in the study results, they were estimated from the Kaplan–Meier survival curves, and the values obtained were utilized in obtaining pooled estimates.
- In this study analysis where PFS and OS rates at different follow-up time points were estimated from the Kaplan–Meier survival curves, an assumption was made that all patients were followed up until this time point. Thus, the denominator for calculation of survival rates was the full sample size. This assumption would not hold true if some patients did not reach this follow-up time point.
- In studies where all adverse events (AEs) observed were reported, any AE not reported in the study was assumed to have an incidence of zero. In studies where all AEs observed were not reported, any AE not reported was considered as missing, since its actual occurrence in patients is unknown.

**Supplementary results**

**Supplementary Table 2** Patient with high-risk features of Hodgkin lymphoma (phase 3 AETHERA study criteria)

| **Study** | **Patient number, n** | **Stage III–IV disease/ Advanced stage, %** | **Primary refractory disease, %** | **Early relapse (relapse with initial remission of <12 months), %** | **Relapse ≥12 months, %** | **B symptoms at relapse, %** | **Extranodal disease at relapse, %** | **Bulky disease at relapse, %** |
| --- | --- | --- | --- | --- | --- | --- | --- | --- |
| **Articles** | | | | | | | |  |
| Flerlage 2019 (1) | 5 | NA | 20 | 20 | NA | NA | NA | NA |
| Sakellari 2019 (2) | 10 | NA | NA | NA | NA | NA | NA | NA |
| Taçyıldız 2019 (3) | 8 | 100 | 37.5 | 37.5 | 25 | NA | NA | NA |
| Kort 2020 (4) | 20 | 60 | 60 | 30 | 10 | 65 | NA | NA |
| Akay 2021 (5) | 75 | NA | 34.6 | 46.7 | 18.7 | 21.3 | 20 | NA |
| Fernandez 2021 (6) | 6 | 83.3 | NA | 100 | NA | NA | NA | 83.3 |
| Kedmi 2021 (7) | 14 | 78.6 | 71.4 | 21.4 | NA | NA | NA | NA |
| Marouf 2022 (8) | 115 | 58 | 43 | 28 | 29 | 17 | 49 | 10 |
| Massano 2022 (9) | 27 | NA | NA | NA | NA | NA | NA | NA |
| Massaro 2022a (10) | 6 | NA | NA^†^ | NA^†^ | NA^†^ | NA | NA | NA |
| Massaro 2022b (11) | 105 | 44 | 48 | 49 | NA | NA | 22 | NA |
| Forlenza 2023 (12) | 67 | 49 | 36 | 64 | 0 | 19 | 35 | 30 |
| Husi 2023 (13) | 108* | NA | NA^†^ | NA^†^ | NA^†^ | NA | NA | NA |
| Martinez 2023 (14) | 62 | 52 | 58 | 26 | NA | 24 | 36 | 3 |
| Wagner 2023 (15) | 118 | 70 | 50 | 29 | 20 | 18 | 39 | NA |
| Damlaj 2023 (16) | 35 | NA^†^ | 54 | 80 | NA | NA | 46 | NA |
| **Abstracts** | | | | | | | |  |
| Aragão 2017 (17) | 8 | NA | NA | 100 | NA | NA | NA | NA |
| Patiño 2019 (18) | 53 | NA | 55 | 28 | NA | NA | 9.4 | NA |
| Chung 2021 (19) | 49 | NA | NA^†^ | NA^†^ | NA^†^ | NA^†^ | NA^†^ | NA^†^ |
| Michalka 2022  (20) | 39 | ~80 | 69^‡^ | NA | NA | NA | NA |  |
| Munoz 2022 (21) | 304 | NA | 7 | NA | NA | NA | NA | NA |
| Falade 2023 (22) | 224 | NA | 79^‡^ | NA | NA | NA^†^ | NA |  |
| Fiad 2024 (23) | 41 | NA* | NA^†^ | NA^†^ | NA | NA | NA^†^ | NA^†^ |

*Exact number of eligible patients not reported. 18 of 126 patients did not receive post-ASCT BV treatment. ^†^Data on high-risk features of Hodgkin lymphoma not reported separately for patients receiving BV as post-ASCT consolidation (study inclusion criteria). ^‡^Primary refractory disease or early relapse (within 12 months after frontline therapy).

ASCT: autologous stem cell transplantation; BV: brentuximab vedotin; NR: not available.

**Supplementary Table 3** Pooled estimates of PFS and OS rates at different time points during follow-up of all patients based on different patient subgroups.

| **Outcomes** | **Number of studies** | **Number of patients** | **Studies** | **Heterogeneity** | | **Pooled estimates, % (95% CI)** |
| --- | --- | --- | --- | --- | --- | --- |
|  |  |  |  | **P value** | ***I^2^, %*** |  |
| **PFS rates** | | | | | | |
| **PFS rates by pre-ASCT BV exposure** | | | | | | |
| ***Pre-ASCT BV naïve patients*** |  |  |  |  |  |  |
| PFS at 1 year | 3 | 247 | Akay 2021 (5)*  Massaro 2022b (11)*  Forlenza 2023 (12) | 0.30 | 18 | 79.8 (71.4–87.2)* |
| PFS at 2 years | 2 | 180 | Akay 2021 (5)  Massaro 2022b (11)* | NC^†^ | NC^†^ | 60.2 (51.0–69.2)* |
| PFS at 3 years | 4 | 309 | Akay 2021 (5)*  Massaro 2022b (11)  Forlenza 2023 (12)  Martinez 2023 (14) | 0.09 | 54 | 66.1 (54.5–76.9)* |
| PFS at 5 years | 1 | 67 | Forlenza 2023 (12) | NC^†^ | NC^†^ | 57.1 (36.5–75.5) |
| ***Pre-ASCT BV exposed patients*** |  |  |  |  |  |  |
| PFS at 1 year | 3 | 247 | Akay 2021 (5)*  Massaro 2022b (11)*  Forlenza 2023 (12) | 0.01 | 78 | 87.1 (69.9–98.1)* |
| PFS at 2 years | 2 | 180 | Akay 2021 (5)  Massaro 2022b (11)* | NC^†^ | NC^†^ | 72.5 (61.2–82.5)* |
| PFS at 3 years | 4 | 309 | Akay 2021 (5)*  Massaro 2022b (11)  Forlenza 2023 (12)  Martinez 2023 (14) | 0.01 | 72 | 84.1 (70.6–94.3)* |
| PFS at 5 years | 1 | 67 | Forlenza 2023 (12) | NC^†^ | NC^†^ | 93.5 (82.5–97.8) |
| **PFS rates by pre-ASCT PET status** | | | | | | |
| ***Patients with positive pre-ASCT PET status*** |  |  |  |  |  |  |
| PFS at 1 year | 1 | 62 | Martinez 2023 (14)* | NC^†^ | NC^†^ | 81.3 (57.0–93.4)* |
| PFS at 2 years | 1 | 62 | Martinez 2023 (14)* | NC^†^ | NC^†^ | 81.3 (57.0–93.4)* |
| PFS at 3 years | 2 | 129 | Forlenza 2023 (12)  Martinez 2023 (14)* | NC^†^ | NC^†^ | 72.3 (56.0–86.2)* |
| ***Patients with negative pre-ASCT PET status*** |  |  |  |  |  |  |
| PFS at 1 year | 1 | 62 | Martinez 2023 (14)* | NC^†^ | NC^†^ | 93.5 (82.5–97.8)* |
| PFS at 2 years | 1 | 62 | Martinez 2023 (14)* | NC^†^ | NC^†^ | 89.1 (77.0–95.3)* |
| PFS at 3 years | 2 | 129 | Forlenza 2023 (12)  Martinez 2023 (14)* | NC^†^ | NC^†^ | 91.1 (84.8–95.9)* |
| PFS at 5 years | 1 | 62 | Martinez 2023 (14) | NC^†^ | NC^†^ | 89.1 (77.0–95.3) |
| **PFS rates by number of risk factors for disease progression** | | | | | | |
| ***Patients with 1–3 risk factors*** |  |  |  |  |  |  |
| PFS at 2 years | 1 | 75 | Akay 2021 (5) | NC^†^ | NC^†^ | 67.2 (55.0–77.4)* |
| ***Patients with 4–5 risk factors*** |  |  |  |  |  |  |
| PFS at 2 years | 1 | 75 | Akay 2021 (5) | NC^†^ | NC^†^ | 72.7 (43.4–90.3)* |
| ***Completed treatment*** |  |  |  |  |  |  |
| PFS at 2 years | 1 | 75 | Akay 2021 (5) | NC^†^ | NC^†^ | 88.1 (75.0–94.8) |
| ***Discontinued treatment*** |  |  |  |  |  |  |
| PFS at 2 years | 1 | 75 | Akay 2021 (5) | NC^†^ | NC^†^ | 82.4 (59.0–93.8) |
| **PFS rates by pre-ASCT response to salvage therapy** | | | | | | |
| ***CR*** |  |  |  |  |  |  |
| PFS at 1 year | 1 | 115 | Marouf 2022 (8) | NC^†^ | NC^†^ | 88.0 (78.7–93.6) |
| PFS at 2 years | 2 | 424 | Marouf 2022 (8)  Munoz 2022 (21) | NC^†^ | NC^†^ | 83.3 (78.6–87.6) |
| PFS at 3 years | 1 | 115 | Marouf 2022 (8) | NC^†^ | NC^†^ | 81.3 (71.1–88.5) |
| PFS at 5 years | 1 | 115 | Marouf 2022 (8) | NC^†^ | NC^†^ | 50.7 (39.6–61.7) |
| ***No CR*** |  |  |  |  |  |  |
| PFS at 1 year | 1 | 115 | Marouf 2022 (8) | NC^†^ | NC^†^ | 68.8 (44.4–85.8) |
| PFS at 2 years | 1 | 424 | Marouf 2022 (8)  Munoz 2022 (21) | NC^†^ | NC^†^ | 50.0 (28.0–72.0) |
| PFS at 3 years | 1 | 115 | Marouf 2022 (8) | NC^†^ | NC^†^ | 50.0 (28.0–72.0) |
| PFS at 5 years | 1 | 115 | Marouf 2022 (8) | NC^†^ | NC^†^ | 50.0 (28.0–72.0) |
| ***PR*** |  |  |  |  |  |  |
| PFS at 2 years | 1 | 309 | Munoz 2022 (21) | NC^†^ | NC^†^ | 57.2 (45.6– 71.8) |
| ***PD*** |  |  |  |  |  |  |
| PFS at 2 years | 1 | 309 | Munoz 2022 (21) | NC^†^ | NC^†^ | 63.6 (45.3–89.5) |
| **OS rates** | | | | | | |
| **OS rates by pre-ASCT BV exposure** | | | | | | |
| ***Pre-ASCT BV naïve patients*** |  |  |  |  |  |  |
| OS at 1 year | 2 | 180 | Akay 2021 (5)*  Massaro 2022b (11)* | NC^†^ | NC^†^ | 94.1 (88.7–97.9)* |
| OS at 2 years | 2 | 180 | Akay 2021 (5)  Massaro 2022b (11)* | NC^†^ | NC^†^ | 85.8 (78.7–91.8)* |
| OS at 3 years | 2 | 180 | Akay 2021 (5)*  Massaro 2022b (11) | NC^†^ | NC^†^ | 70.1 (61.2–78.3)* |
| ***Pre-ASCT BV exposed patients*** |  |  |  |  |  |  |
| OS at 1 year | 2 | 180 | Akay 2021 (5)*  Massaro 2022b (11)* | NC^†^ | NC^†^ | 96.4 (90.0–99.9)* |
| OS at 2 years | 2 | 180 | Akay 2021 (5)  Massaro 2022b (11)* | NC^†^ | NC^†^ | 96.4 (90.0–99.9)* |
| OS at 3 years | 2 | 180 | Akay 2021 (5)*  Massaro 2022b (11) | NC^†^ | NC^†^ | 96.4 (90.0–99.9)* |
| **OS rates by completion of BV consolidation therapy** | | | | | | |
| ***Completed treatment*** |  |  |  |  |  |  |
| OS at 2 years | 1 | 75 | Akay 2021 (5) | NC^†^ | NC^†^ | 100.0 (91.6–100.0) |
| ***Discontinued treatment*** |  |  |  |  |  |  |
| OS at 2 years | 1 | 75 | Akay 2021 (5) | NC^†^ | NC^†^ | 94.1 (73.0–99.0) |

*PFS/OS rates were estimated from Kaplan–Meier curves or pooled estimates of PFS/OS rates reported/estimated from Kaplan–Meier curves from 2 or more studies. ^†^Heterogeneity was not computed for outcomes with data provided by only 1 or 2 studies.

ASCT: autologous stem cell transplantation; BV: brentuximab vedotin; CI: confidence interval; CR: complete response; NC: not computed; OS: overall survival; PET: positron emission tomography; PFS: progression-free survival; PR: partial response.

**Supplementary Table 4** Pooled incidence of individual any grade AEs in patients with RRHL treatment with BV as post-ASCT consolidation*

| **Outcomes** | **Number of studies** | **Number of patients** | **Studies** | **Heterogeneity** | | **Pooled % (95% CI)** |
| --- | --- | --- | --- | --- | --- | --- |
|  |  |  |  | **P value** | ***I^2^, %*** |  |
| Neuropathy | 14 | 751 | Aragão 2017 (17)  Flerlage 2019 (1)  Patiño 2019 (18)  Akay 2021 (5)  Chung 2021 (19)  Fernandez 2021 (6)  Kedmi 2021 (7)  Marouf 2022 (8)  Massaro 2022b (11)  Michalka 2022 (20)  Damlaj 2023 (16)  Forlenza 2023 (12)  Martinez 2023 (14)  Wagner 2023 (15) | <0.001 | 92 | 34.2 (21.8–47.6)* |
| Neutropenia | 11 | 532 | Flerlage 2019 (1)  Akay 2021 (5)  Fernandez 2021 (6)  Kedmi 2021 (7)  Massaro 2022a (10)  Massaro 2022b (11)  Michalka 2022 (20)  Damlaj 2023 (16)  Forlenza 2023 (12)  Martinez 2023 (14)  Wagner 2023 (15) | <0.001 | 92 | 20.2 (7.6–36.2)* |
| Infections | 8 | 452 | Flerlage 2019 (1)  Akay 2021 (5)  Fernandez 2021 (6)  Kedmi 2021 (7)  Massaro 2022b (11)  Forlenza 2023 (12)  Martinez 2023 (14)  Wagner 2023 (15) | <0.001 | 84 | 2.7 (0.0–10.0)* |
| Thrombocytopenia | 7 | 272 | Flerlage 2019 (1)  Akay 2021 (5)  Fernandez 2021 (6)  Kedmi 2021 (7)  Massaro 2022b (11)  Forlenza 2023 (12)  Martinez 2023 (14) | 0.01 | 64 | 1.3 (0.0–6.0)* |
| Anemia | 8 | 282 | Flerlage 2019 (1)  Sakellari 2019 (2)  Akay 2021 (5)  Fernandez 2021 (6)  Kedmi 2021 (7)  Massaro 2022b (11)  Forlenza 2023 (12)  Martinez 2023 (14) | 0.05 | 50 | 1.0 (0.0–5.0)* |
| Fatigue | 7 | 325 | Flerlage 2019 (1)  Patiño 2019 (18)  Akay 2021 (5)  Fernandez 2021 (6)  Kedmi 2021 (7)  Massaro 2022b (11)  Forlenza 2023 (12) | 0.01 | 65 | 0.7 (0.0–5.0)* |
| Nausea/vomiting | 7 | 334 | Flerlage 2019 (1)  Akay 2021 (5)  Fernandez 2021 (6)  Kedmi 2021 (7)  Massaro 2022b (11)  Forlenza 2023 (12)  Martinez 2023 (14) | 0.01 | 65 | 0.6 (0.0–5.0)* |
| Pulmonary toxicity | 6 | 272 | Flerlage 2019 (1)  Akay 2021 (5)  Fernandez 2021 (6)  Kedmi 2021 (7)  Massaro 2022b (11)  Forlenza 2023 (12) | 0.04 | 58 | 0.5 (0.0–5.0)* |
| Transaminitis | 6 | 272 | Flerlage 2019 (1)  Akay 2021 (5)  Fernandez 2021 (6)  Kedmi 2021 (7)  Massaro 2022b (11)  Forlenza 2023 (12) | 0.08 | 50 | 0.3 (0.0–4.0)* |
| Infusion reaction | 6 | 272 | Flerlage 2019 (1)  Akay 2021 (5)  Fernandez 2021 (6)  Kedmi 2021 (7)  Massaro 2022b (11)  Forlenza 2023 (12) | 0.31 | 16 | 0.1 (0.0–2.1)* |
| Pneumonia | 7 | 334 | Flerlage 2019 (1)  Akay 2021 (5)  Fernandez 2021 (6)  Kedmi 2021 (7)  Massaro 2022b (11)  Forlenza 2023 (12)  Martinez 2023 (14) | 0.15 | 36 | 0.0 (0.0–1.7)* |
| Respiratory tract infection | 6 | 272 | Flerlage 2019 (1)  Akay 2021 (5)  Fernandez 2021 (6)  Kedmi 2021 (7)  Massaro 2022b (11)  Forlenza 2023 (12) | 0.30 | 17 | 0.0 (0.0–0.7)* |
| Cough | 6 | 272 | Flerlage 2019 (1)  Akay 2021 (5)  Fernandez 2021 (6)  Kedmi 2021 (7)  Massaro 2022b (11)  Forlenza 2023 (12) | 0.54 | 0 | 0.0 (0.0–0.2)* |
| Secondary malignancy | 7 | 292 | Flerlage 2019 (1)  Kort 2020 (4)  Akay 2021 (5)  Fernandez 2021 (6)  Kedmi 2021 (7)  Massaro 2022b (11)  Forlenza 2023 (12) | 0.53 | 0 | 0.0 (0.0–0.2)* |
| Constipation | 6 | 272 | Flerlage 2019 (1)  Akay 2021 (5)  Fernandez 2021 (6)  Kedmi 2021 (7)  Massaro 2022b (11)  Forlenza 2023 (12) | 0.80 | 0 | 0.0 (0.0–0.1)* |
| Acute bronchitis | 6 | 272 | Flerlage 2019 (1)  Akay 2021 (5)  Fernandez 2021 (6)  Kedmi 2021 (7)  Massaro 2022b (11)  Forlenza 2023 (12) | 0.80 | 0 | 0.0 (0.0–0.1)* |
| Otitis media | 6 | 272 | Flerlage 2019 (1)  Akay 2021 (5)  Fernandez 2021 (6)  Kedmi 2021 (7)  Massaro 2022b (11)  Forlenza 2023 (12) | 0.80 | 0 | 0.0 (0.0–0.1)* |
| Arthralgia | 8 | 340 | Flerlage 2019 (1)  Akay 2021 (5)  Fernandez 2021 (6)  Kedmi 2021 (7)  Forlenza 2023 (12)  Massaro 2022a (10)  Massaro 2022b (11)  Martinez 2023 (14) | 0.45 | 0 | 0.0 (0.0–0.1)* |

*This table summarizes the common adverse events reported by 2 or more studies, indicating the occurrence of 1 or more instances (non-zero) for each adverse event.

AE: adverse event; ASCT: autologous stem cell transplantation; BV: brentuximab vedotin; CI: confidence interval; RRHL: relapsed/refractory Hodgkin lymphoma.

**Supplementary Fig. 1** Pooled estimates of PFS rates at 1 and 11 years of follow-up in all patients based on PFS definitions

**a** PFS rate at 1 year of follow-up


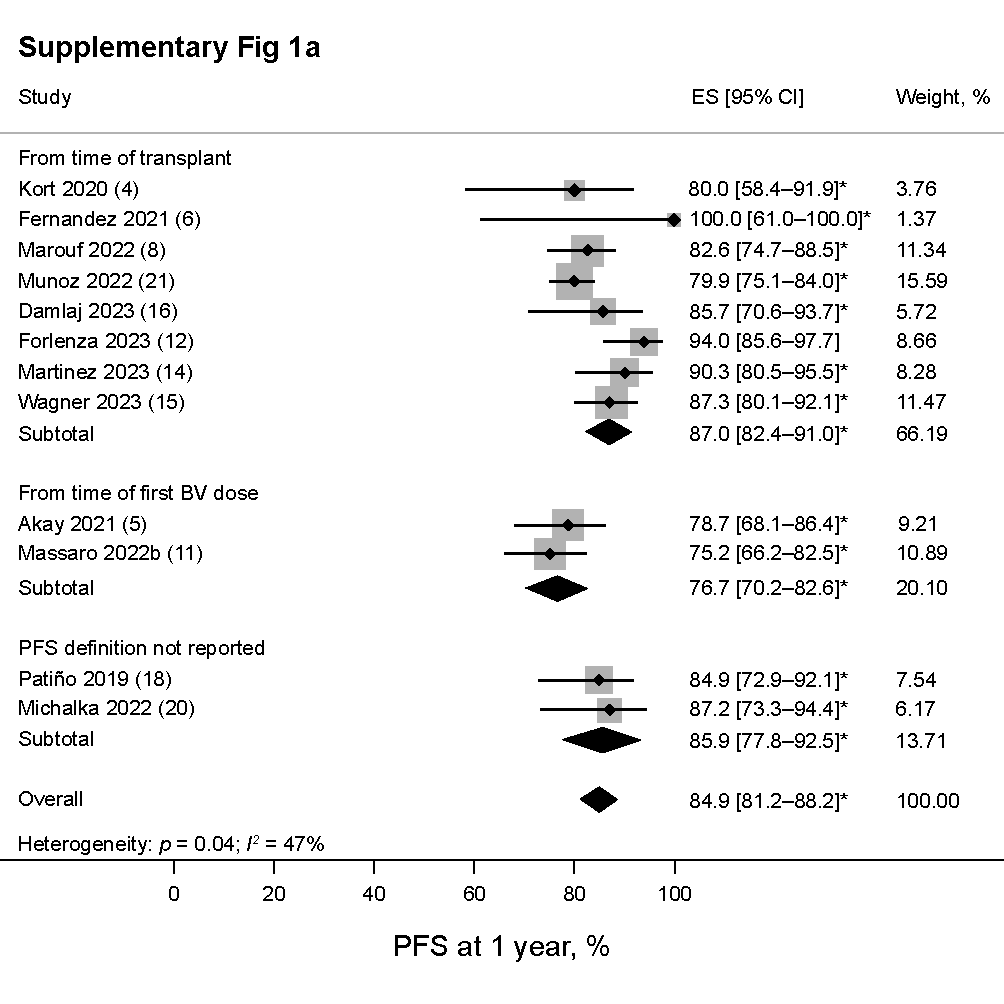


**b** PFS rates at 11 years of follow-up


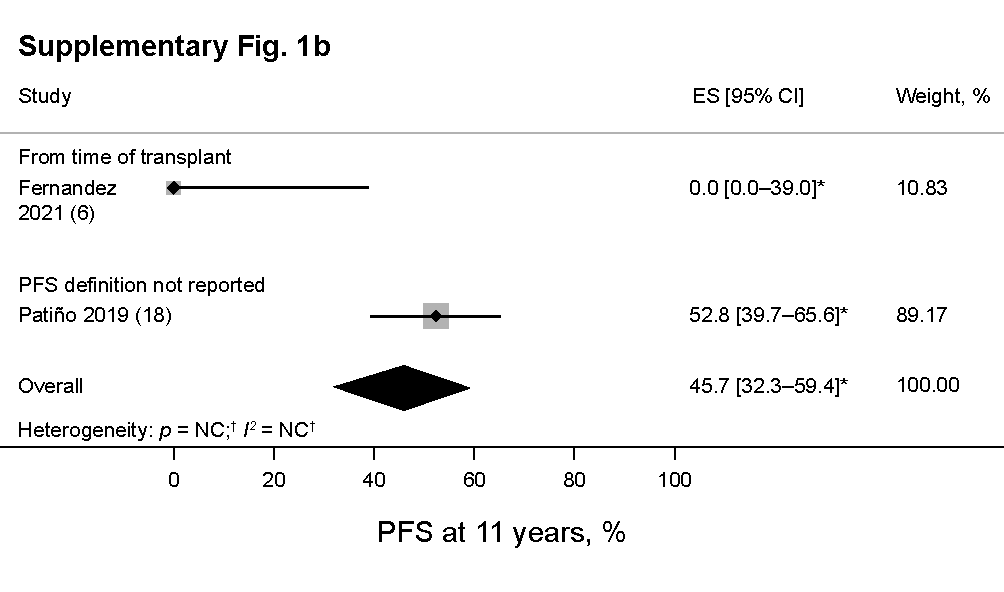


^*^PFS rates were estimated using Kaplan–Meier curves. ^†^Heterogeneity was not computed for outcomes with data provided by only 1 or 2 studies.

CI: confidence interval; ES: effect size; NC: not calculated; PFS: progression-free survival.

**Supplementary Fig. 2** Pooled estimates of OS rates at 1 and 11 years of follow-up in all patients based on OS definitions

**a** OS rates at 1 year of follow-up


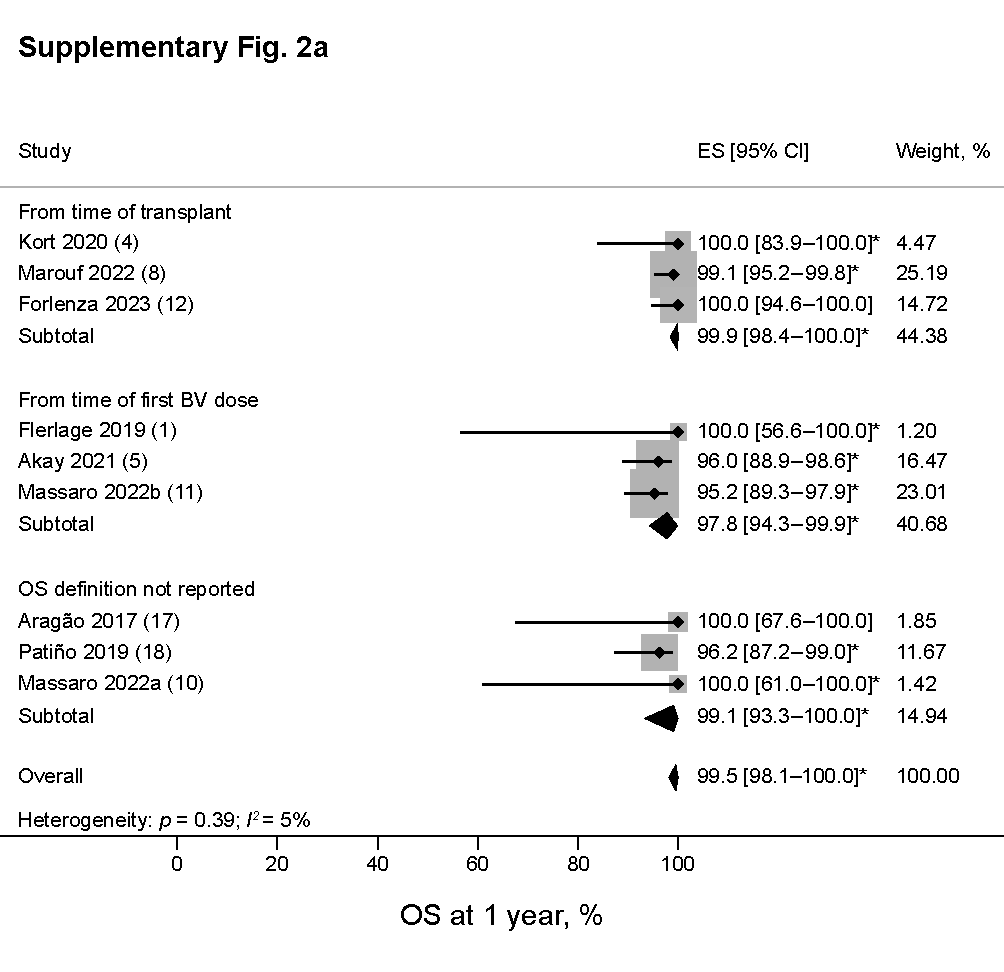


**b** OS rates at 11 years of follow-up


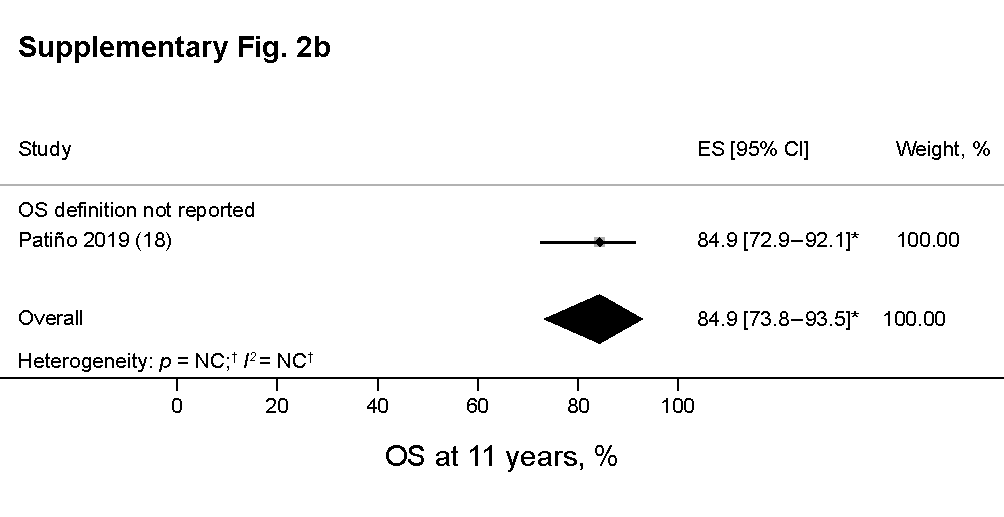


*OS rates were estimated using Kaplan–Meier curves.

CI: confidence interval; ES: effect size; NC: not calculated; OS: overall survival.

**Supplementary Fig. 3.** Pooled estimates of AEs in patients with RRHL treatment with BV as post-ASCT consolidation.* Mean number of **a** any grade AEs per patient **b** Grade 3–4 AEs per patient; incidence of **c** ≥1 any grade and **d** ≥1 Grade 3–4 AEs; incidence of (**e**) any grade neuropathy and (**f**) any grade neutropenia

**a** Mean any grade AEs per patient


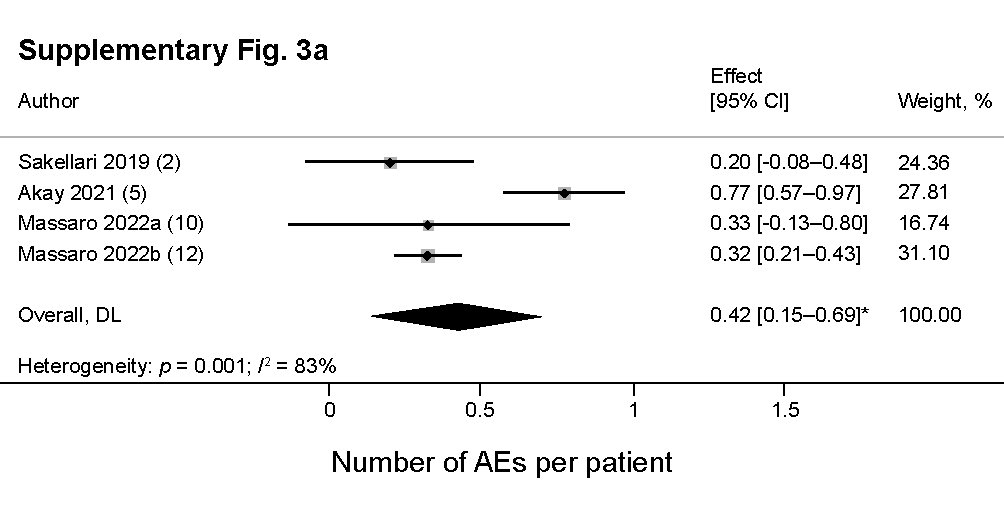


**b** Mean Grade 3–4 AEs per patient


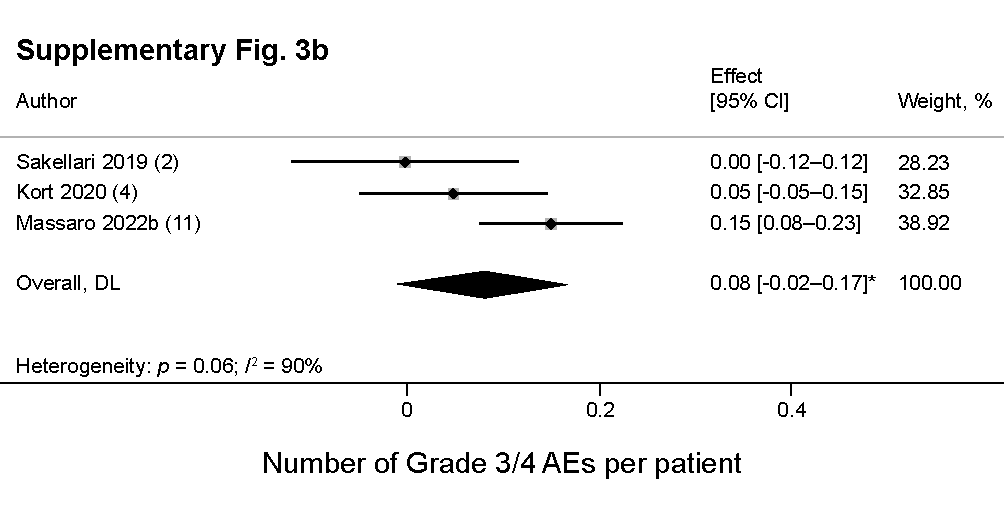


**c** Proportion of ≥1 any grade AE


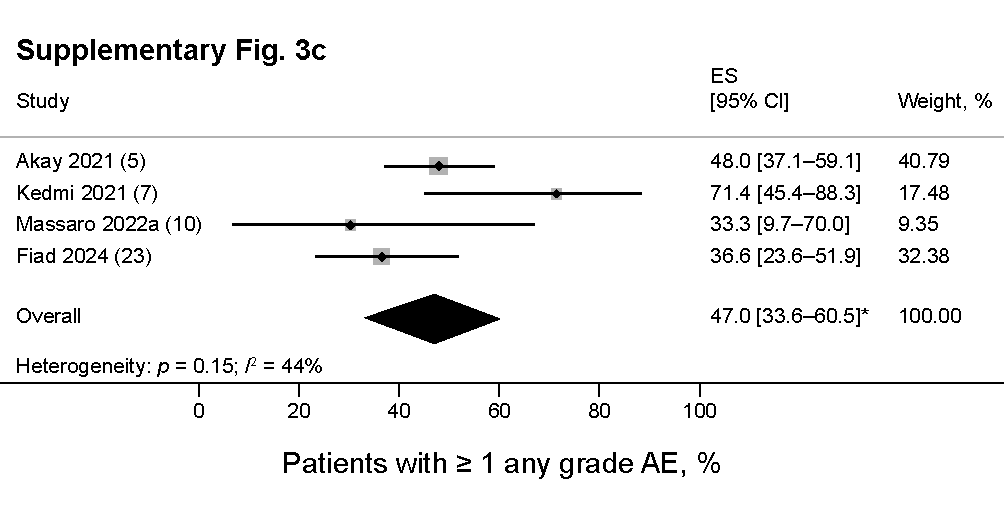


**d** Proportion of ≥1 Grade 3–4 AE

**
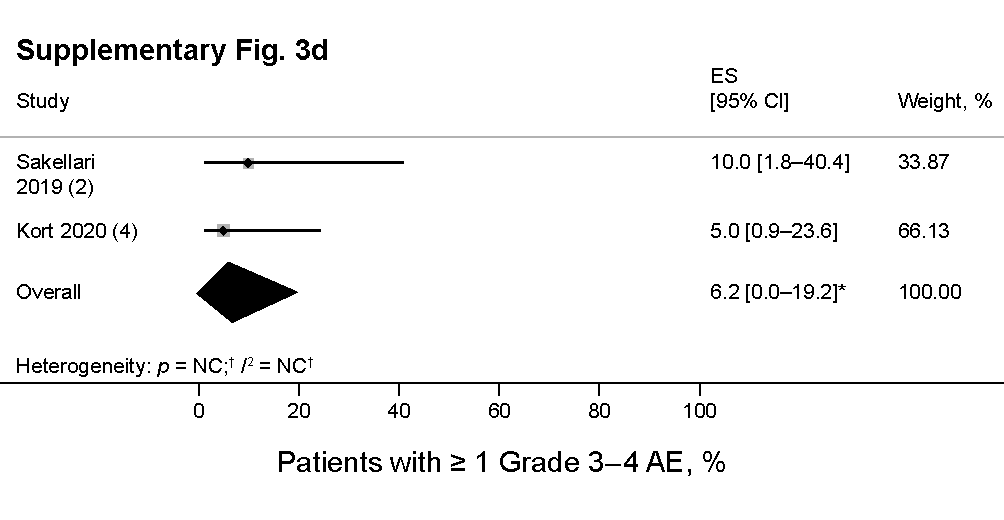
**

**e** Incidence of any grade neuropathy


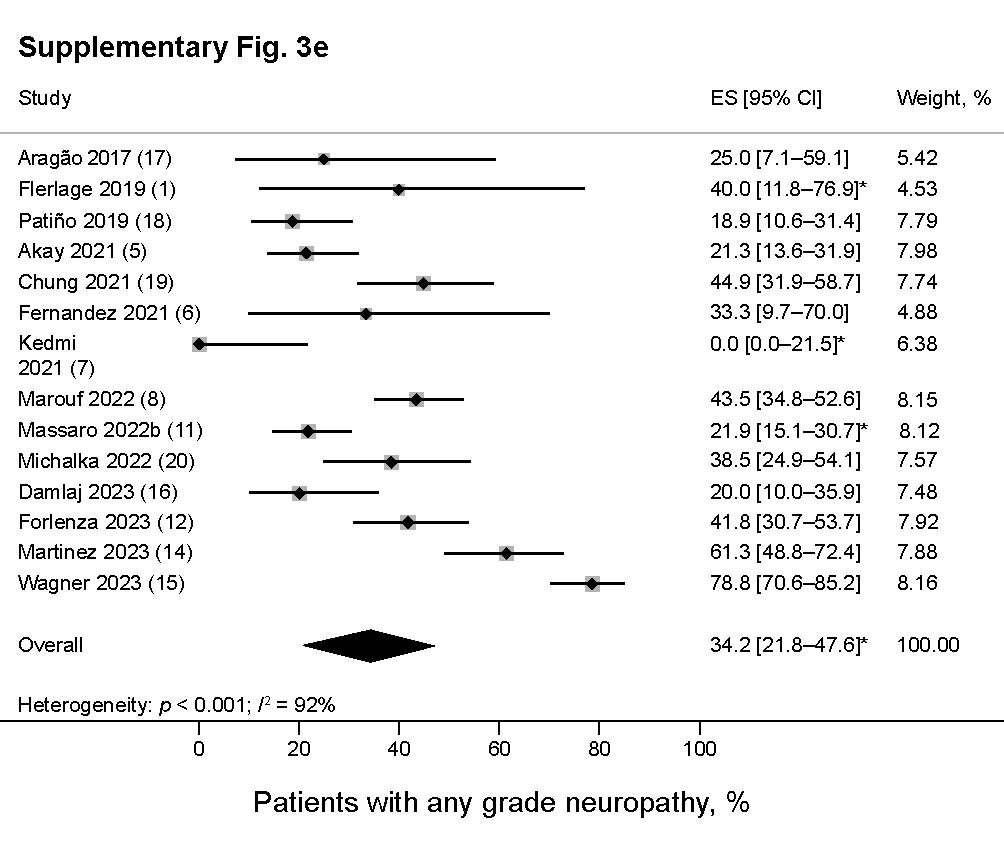


**f** Incidence of any grade neutropenia


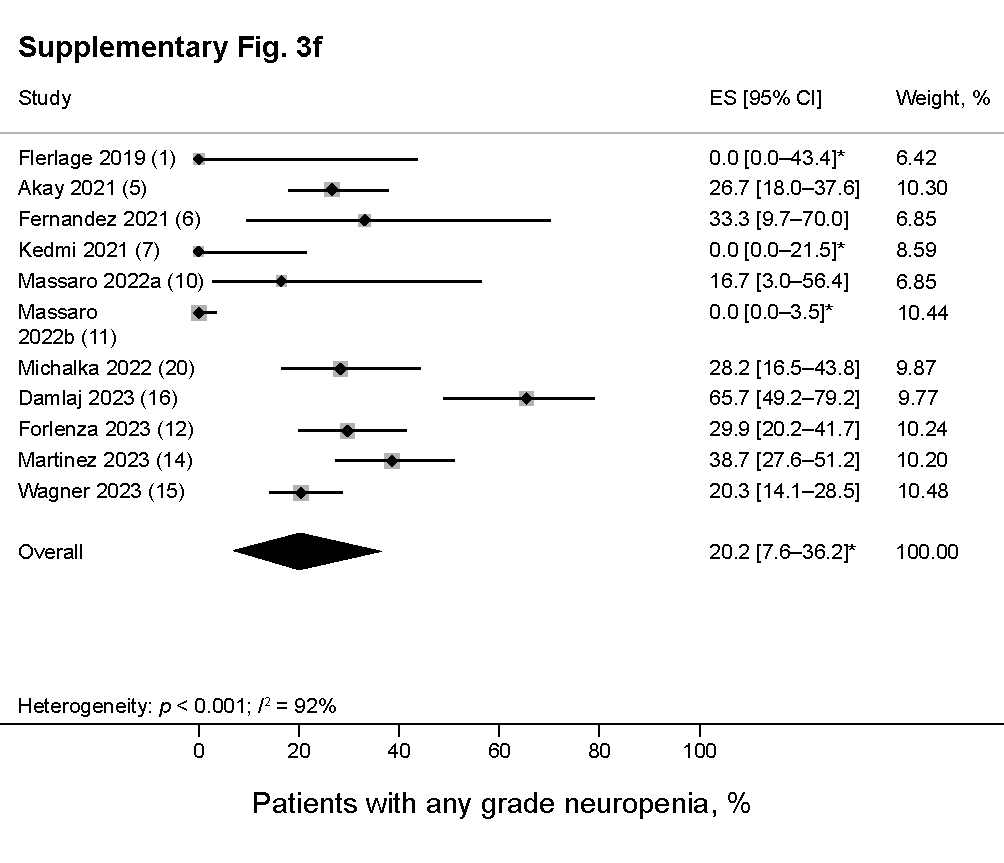
*Estimated. AE: adverse event; ASCT: autologous stem cell transplantation; BV: brentuximab vedotin; CI: confidence interval; ES: effect size; NC: not calculated; RRHL: relapsed/refractory Hodgkin lymphoma.

**References**

1. Flerlage JE, von Buttlar X, Krasin M, Triplett B, Kaste SC, Metzger ML. Brentuximab vedotin as consolidation after hematopoietic cell transplant for relapsed Hodgkin lymphoma in pediatric patients. Pediatr Blood Cancer. 2019;66(12):e27962.

2. Sakellari I, Gavriilaki E, Iskas M, Bousiou Z, Chatziioannidis A, Batsis I, et al. Brentuximab vedotin and anti-PD1 treatment optimize survival in chemo-refractory Hodgkin lymphoma patients: Real-world data. Hematol Oncol. 2019;37(4):490–2.

3. Tacyildiz N, Tanyildiz HG, Unal E, Dincaslan H, Asarcikli F, Aksoy BA, et al. A targeted salvage therapy with Brentuximab vedotin in heavily treated refractory or relapsed pediatric Hodgkin lymphoma patients before and after stem cell transplantation. Turk J Pediatr. 2019;61(5):671–6.

4. Kort J, Chidiac A, El Sayed R, Massoud R, Nehme R, Bazarbachi A, et al. Safety and efficacy of four cycles of brentuximab vedotin as consolidation after autologous peripheral stem cell transplantation in relapsed/refractory Hodgkin lymphoma. Leuk Lymphoma. 2020;61(7):1732–5.

5. Akay OM, Ozbalak M, Pehlivan M, Yildiz B, Uzay A, Yigenoglu TN, et al. Brentuximab vedotin consolidation therapy after autologous stem-cell transplantation in patients with high-risk Hodgkin lymphoma: Multicenter retrospective study. Hematol Oncol. 2021;39(4):498–505.

6. Fernandez KS, Mavers M, Marks LJ, Agarwal R. Brentuximab vedotin as consolidation therapy after autologous stem cell transplantation in children and adolescents (<18 y) with early relapse Hodgkin lymphoma. J Pediatr Hematol Oncol. 2021;43(2):e191–e4.

7. Kedmi M, Khaustov P, Ribakovsy E, Benjamini O, Avigdor A. Outcomes related to FDG-PET-CT response in patients With Hodgkin lymphoma treated with brentuximab-vedotin at relapse or consolidation. Clin Lymphoma Myeloma Leuk. 2021;21(12):e929–e37.

8. Marouf A, Cottereau AS, Kanoun S, Deschamps P, Meignan M, Franchi P, et al. Outcomes of refractory or relapsed Hodgkin lymphoma patients with post-autologous stem cell transplantation brentuximab vedotin maintenance: a French multicenter observational cohort study. Haematologica. 2022;107(7):1681–6.

9. Massano D, Carraro E, Mussolin L, Buffardi S, Barat V, Zama D, et al. Brentuximab vedotin in the treatment of paediatric patients with relapsed or refractory Hodgkin's lymphoma: Results of a real-life study. Pediatr Blood Cancer. 2022;69(10):e29801.

10. Massaro F, Meuleman N, Bron D, Vercruyssen M, Maerevoet M. Brentuximab vedotin and pembrolizumab combination in patients with relapsed/refractory Hodgkin lymphoma: A single-centre retrospective analysis. Cancers (Basel). 2022;14(4).

11. Massaro F, Pavone V, Stefani PM, Botto B, Pulsoni A, Patti C, et al. Brentuximab vedotin consolidation after autologous stem cell transplantation for Hodgkin lymphoma: A Fondazione Italiana Linfomi real-life experience. Hematol Oncol. 2022;40(1):31–9.

12. Forlenza CJ, Rosenzweig J, Mauguen A, Buhtoiarov I, Cuglievan B, Dave H, et al. Brentuximab vedotin after autologous transplantation in pediatric patients with relapsed/refractory Hodgkin lymphoma. Blood Adv. 2023;7(13):3225–31.

13. Husi K, Szabo R, Pinczes LI, Foldeak D, Dudley R, Szomor A, et al. Improved survival of autologous stem cell transplantation in primary refractory and relapsed Hodgkin lymphoma in the brentuximab vedotin era - real-world data from Hungary. Ann Hematol. 2023;102(9):2555–63.

14. Martinez C, de Haro ME, Romero S, Gutierrez A, Domingo-Domenech E, Gonzalez-Rodriguez AP, et al. Impact of pre- and/or post-autologous stem cell transplantation exposure to brentuximab vedotin on survival outcomes in patients with high-risk Hodgkin lymphoma. Ann Hematol. 2023;102(2):429–37.

15. Wagner CB, Boucher K, Nedved A, Micallef IN, Desai S, Hatic H, et al. Effect of cumulative dose of brentuximab vedotin maintenance in relapsed/refractory classical Hodgkin lymphoma after autologous stem cell transplant: an analysis of real-world outcomes. Haematologica. 2023;108(11):3025–32.

16. Damlaj M, Tlayjeh M, Damlaj A, Alahmari B, AlSadi H, Ahmed M, et al. Contemporary outcomes of high risk relapsed refractory classical hodgkin lymphoma patients-role of maintenance therapy in the real world. Bone Marrow Transplant. 2023;58(10):1160-2.

17. Aragão L, Bispo O, Carvalho H, Silva A, Souza V, Carvalho L, et al. Brentuximab vedotin consolidation therapy after autologous stem-cell transplantation in patients with high-risk Hodgkin’s lymphoma: The real world experience of a single bone marrow transplant center in Salvador/Bahia. Clin Lymphoma Myeloma Leuk. 2017;17:S324.

18. Patiño B, Acon-Solano C, Pereira M, Enciso-Olivera L, Hoz DO-dl, Cordero HM, et al. Brentuximab vedotin as consolidation therapy post hematopoietic stem cell transplantation for patients with relapsed or refractory Hodgkin lymphoma: A real world analysis of the Colombian experience. Blood. 2019;134:5275.

19. Chung S, White J, Toze CL, Sutherland HJ, Sanford D, Rodrigo JA, et al. Autologous stem cell transplant outcome in classic Hodgkin lymphoma patients in the era of post-transplant brentuximab vedotin consolidation. Blood. 2021;138:3935.

20. Michalka J, Marková J, Gahérová Ľ, Maco M, Procházka V, Kořen J, et al. P104: Consolidation therapy with brentuximab vedotin after autologous stem cell transplantation for relapsed/refractory Hodgkin lymphoma in the Czech Republic. HemaSphere. 2022;6:48–9.

21. Munoz MCM, Bastos-Oreiro M, Boumendil A, Finel H, Bazarbachi A, Alzahrani M, et al. Real life analysis of brentuximab vedotin (bv) use as consolidation therapy in patients with Hodgkin's lymphoma (hl) with high risk of relapse after autologous stem cell transplantation (asct). a retrospective analysis of the EBMT Lymphoma Working Party. Blood. 2022;140:6558–60.

22. Falade AS, Redd RA, Shah H, Baron K, Iyengar S, Desai SH, et al. Efficacy of brentuximab vedotin maintenance therapy following autologous stem cell transplantation in patients with relapsed/refractory classical Hodgkin lymphoma with and without pre-transplant exposure to novel agents. Blood. 2023;142:3062.

23. Fiad L, Warley F, Altuve JG, Cerutti A, Salvano L, Guanchiale L, et al. Prospective real-world evidence of brentuximab vedotin (BV) consolidation after autologous hematopoietic stem cell transplantation (ASCT) in patients with relapsed/refractory Hodgkin lymphoma (RR HL). HemaSphere. 2024.
